# Neurovascular dysfunction and neuroinflammation in a Cockayne syndrome mouse model

**DOI:** 10.18632/aging.203617

**Published:** 2021-10-10

**Authors:** Gustavo Satoru Kajitani, Lear Brace, Jose Humberto Trevino-Villarreal, Kaspar Trocha, Michael Robert MacArthur, Sarah Vose, Dorathy Vargas, Roderick Bronson, Sarah Jayne Mitchell, Carlos Frederico Martins Menck, James Robert Mitchell

**Affiliations:** 1 Department of Genetics and Complex Diseases, Harvard School of Public Health, Boston, MA 02115, USA; 2 Departamento de Microbiologia, Instituto de Ciências Biomédicas, Universidade de São Paulo, São Paulo, Brazil; 3 Department of Health Sciences and Technology, Swiss Federal Institute of Technology (ETH) Zurich, Zurich, Switzerland; 4 Rodent Histopathology Core, Department of Pathology, Harvard Medical School, Boston, MA 02115, USA

**Keywords:** Cockayne syndrome, CSA, XPA, segmental progeria, inflammation, vascular dysfunction

## Abstract

Cockayne syndrome (CS) is a rare, autosomal genetic disorder characterized by premature aging-like features, such as cachectic dwarfism, retinal atrophy, and progressive neurodegeneration. The molecular defect in CS lies in genes associated with the transcription-coupled branch of the nucleotide excision DNA repair (NER) pathway, though it is not yet clear how DNA repair deficiency leads to the multiorgan dysfunction symptoms of CS. In this work, we used a mouse model of severe CS with complete loss of NER (*Csa−/−/Xpa−/−*), which recapitulates several CS-related phenotypes, resulting in premature death of these mice at approximately 20 weeks of age. Although this CS model exhibits a severe progeroid phenotype, we found no evidence of *in vitro* endothelial cell dysfunction, as assessed by measuring population doubling time, migration capacity, and ICAM-1 expression. Furthermore, aortas from CX mice did not exhibit early senescence nor reduced angiogenesis capacity. Despite these observations, CX mice presented blood brain barrier disruption and increased senescence of brain endothelial cells. This was accompanied by an upregulation of inflammatory markers in the brains of CX mice, such as ICAM-1, TNFα, p-p65, and glial cell activation. Inhibition of neovascularization did not exacerbate neither astro- nor microgliosis, suggesting that the pro-inflammatory phenotype is independent of the neurovascular dysfunction present in CX mice. These findings have implications for the etiology of this disease and could contribute to the study of novel therapeutic targets for treating Cockayne syndrome patients.

## INTRODUCTION

The DNA molecule is under constant physical and chemical stress which can result in DNA damage [1]. These lesions, if not correctly repaired, may induce several biological processes which can be detrimental to organismal health. Notably, DNA damage and its repair have been implicated in the process of aging. DNA damage accumulates with age and can trigger processes such as senescence, cell signaling alterations and cell death, all of which are associated with pathologies of aging, including brain ageing [1–4]. Furthermore, dysfunctional DNA repair genes may lead to genetic diseases characterized by segmental progeroid features, such as altered endocrine axes and tissue degeneration [5]. These diseases are often associated with accelerated aging of the brain, including progressive and profound neurodegeneration [6, 7]. There are numerous genetic syndromes associated with DNA repair deficiencies, particularly affecting nucleotide excision repair (NER), including Cockayne syndrome (CS), Trichotiodystrophy (TTD), and xeroderma pigmentosum (XP) [8, 9].

Cockayne syndrome is a rare, autosomal, recessive disorder caused by mutations in one of two genes (*CSA* or *CSB*) involved in the transcription-coupled nucleotide excision DNA repair (TC-NER) pathway. CS is characterized by a wide range of symptoms, including cachectic dwarfism, lipodystrophy, photosensitivity, multiorgan degeneration, and early mortality [10]. Regarding neurodegeneration in CS, various neurological abnormalities are observed such as loss of Purkinje cells in the cerebellum, reduced numbers of oligodendrocytes, demyelination of central and peripheral nervous tissue, brain calcification, and microcephaly [6]. It is worth noting that although CS has been extensively studied, the mechanisms underlying the progeroid/neurodegenerative phenotype are not yet fully understood, and there are currently no therapies for CS patients [11].

Various knockout mouse models to study the relationship between DNA repair deficiency and progeroid syndromes have been developed and characterized. These include the *Ercc1*^−/Δ^ [12], *Xpd/Xpa* [13], *Csb^m/m^* [14], *Csb^m/m^/Xpa*^−*/*−^ [15], and *Csa*^−*/*−^*/Xpa*^−*/*−^ [16] models. These models exhibit a spectrum of phenotypes that mimic those of human CS, such as reduced weight and size, indicating postnatal developmental defects, progressive loss of adiposity, kyphosis, abnormal gait, hindlimb paralysis, and premature death [16]. Other notable neurological symptoms in these mouse models often include reduced cerebellar size and decreased white matter, loss of Purkinje cells, and patchy areas of myelin loss [17]. Although these models show broad similarities, there are some differences among them. For instance, *Ercc1^−/Δ^* fibroblasts exhibit early senescence, while *Xpd/Xpa* fibroblasts do not [12, 13].

Besides neuron-specific DNA repair defects [17], other factors have been implicated in CS-associated neuropathology. Vascular dysfunction and neuroinflammation have been proposed as causes of the progressive neurodegeneration of CS, as both of these pathologic processes were found in CS patients and progeroid mouse models [18–20]. Endothelial cells (ECs) form the inner lining of blood vessels and have essential roles in every organ, including the brain, where they are responsible for processes such as the formation and maintenance of the blood brain barrier (BBB), energy metabolism, and inflammation [21, 22]. In addition to ECs, other cell types play a role in brain inflammation including neurons, astrocytes and microglia [23–26]. The oxidative stress generated by a neuroinflammatory response can result in myelin abnormalities and neuronal cell death, as lipid peroxidation products stemming from myelin oxidation can damage neuronal DNA, resulting in activation of pro-apoptotic pathways [6]. There is evidence that neuroinflammation also contributes to other aspects of neurodegenerative disorders, including altered neuron morphology and synapse elimination [23–26]. Besides neuronal cell death, DNA lesions can also play a role in neuroinflammation, as DNA damage can result in mitophagy impairment through a PARP1-dependent signaling [27]. Loss of mitochondrial homeostasis has been found to induce not only alterations in cellular metabolism, but also activate pro-inflammatory pathways through damaged-associated molecular patterns [4].

In this work, we show that the *Csa*^−*/*−^*/Xpa*^−*/*−^ (herein referred to as CX) mouse model presents neurological dysfunction resembling CS patients, including myelin loss and loss of Purkinje cells. Furthermore, although we found no evidence of cell autonomous EC dysfunction *in vitro* using cell proliferation, wound healing, ICAM-1 activation and angiogenesis assays, CX mice presented numerous *in vivo* markers of brain vascular dysfunction. These include increased brain vascular permeability, vascular activation/inflammation gene expression, EC senescence, upregulation of active NF-κB, and an increase in active astrocytes and microglia.

These results indicate that neuroinflammation is likely a factor contributing to the neuropathology of these animals *in vivo*, providing a novel insight into the etiology of this complex and devastating disease.

## RESULTS

### Central nervous system dysfunction and brain DNA damage in aged CX mice

Csa^−*/*−^/Xpa^−*/*−^ mice were previously reported to have several functional neurological impairments, including abnormal gait, inappropriate hind limb clasping, reduced grip strength, and dystonia, leading to eventual paralysis [16]. The maximum lifespan of these mice is on average 20 weeks [16, 28]. To investigate the central nervous system dysfunction in CX mice, we first performed histological analyses in young pre-weaning mice and older 17-week-old mice.

**Figure 1:**
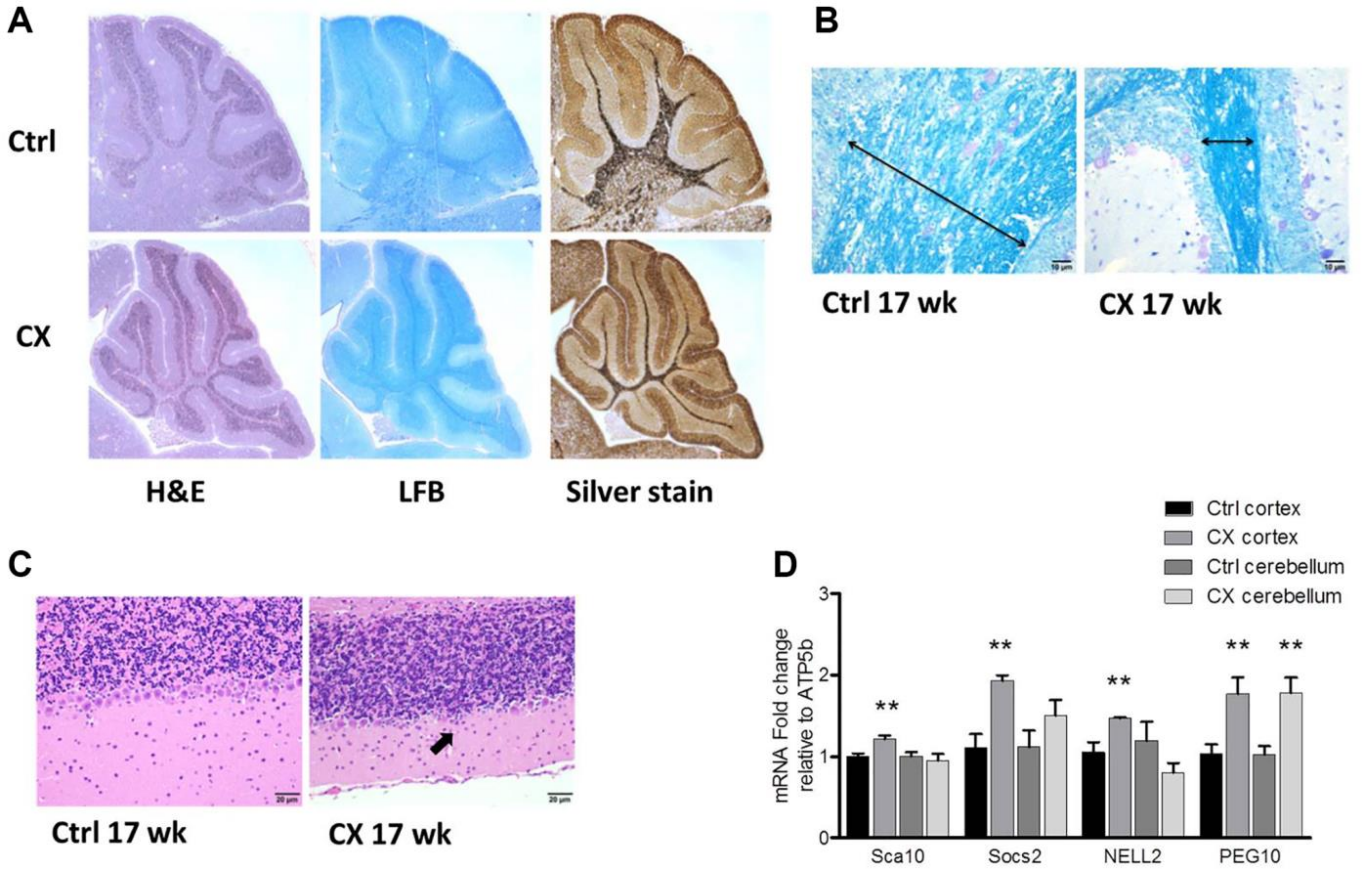
**Neurodegeneration and myelin loss in CX mice.** (**A**) Hematoxylin and Eosin (H&E), Luxol fast blue (LFB), and silver staining of cerebellum of pre-weaning control and CX animals. (**B**) Luxol fast blue staining of cerebellum from 17-week old control and CX animals. Myelin content length indicated by arrow. (**C**) Hematoxylin and Eosin staining of the Purkinje cell layer of animals described in b. CX mice display degeneration of the Purkinje cell layer (arrow). *n* = 2. (**D**) Gene expression of control and CX cortex and cerebellum for PARP-1 regulated genes, *n* = 8. Data are presented as mean ± SE. Student’s *t*-test. ^*^P < 0.05, ^**^P < 0.01. Abbreviations: Ctrl: control; WT: wildtype; Wk: weeks.

Myelin loss was detected both in pre-weaning and 17-week-old CX mice, as evidenced by a decrease in white matter and luxol fast blue staining of lipids (Figure 1A–1B, Supplementary Figure 1). Increased silver staining of argyrophillic neurons was observed in pre-weaning CX mice (Figure 1A), indicating neuronal degeneration. Accordingly, the Purkinje cell layer was reduced in the cerebellum of 17 week old CX mice (Figure 1C, Supplementary Figure 1), as often observed in CS patients [11]. Surprisingly, activation of DNA damage response was found mainly in the cortex of aged CX mice, as mRNA expression of several PARP-1 regulated genes (Sca10, Socs2, Nell2 and Peg10) [29] was significantly increased in CX cortex, but not in the cerebellum, with the exception of Peg10, which was increased in both brain regions (Figure 1D). Furthermore, the spinal cord and sciatic nerve of CX mice also showed histological abnormalities, especially myelin sheath degeneration (Supplementary Figure 2). CX nervous tissue also presented higher fatty acid oxidation capacity and increased expression of fatty acid oxidation-related genes (Supplementary Figure 3), in accordance to previous findings [28].

### Blood brain barrier dysfunction and endothelial cell activation in CX mice

**Figure 2:**
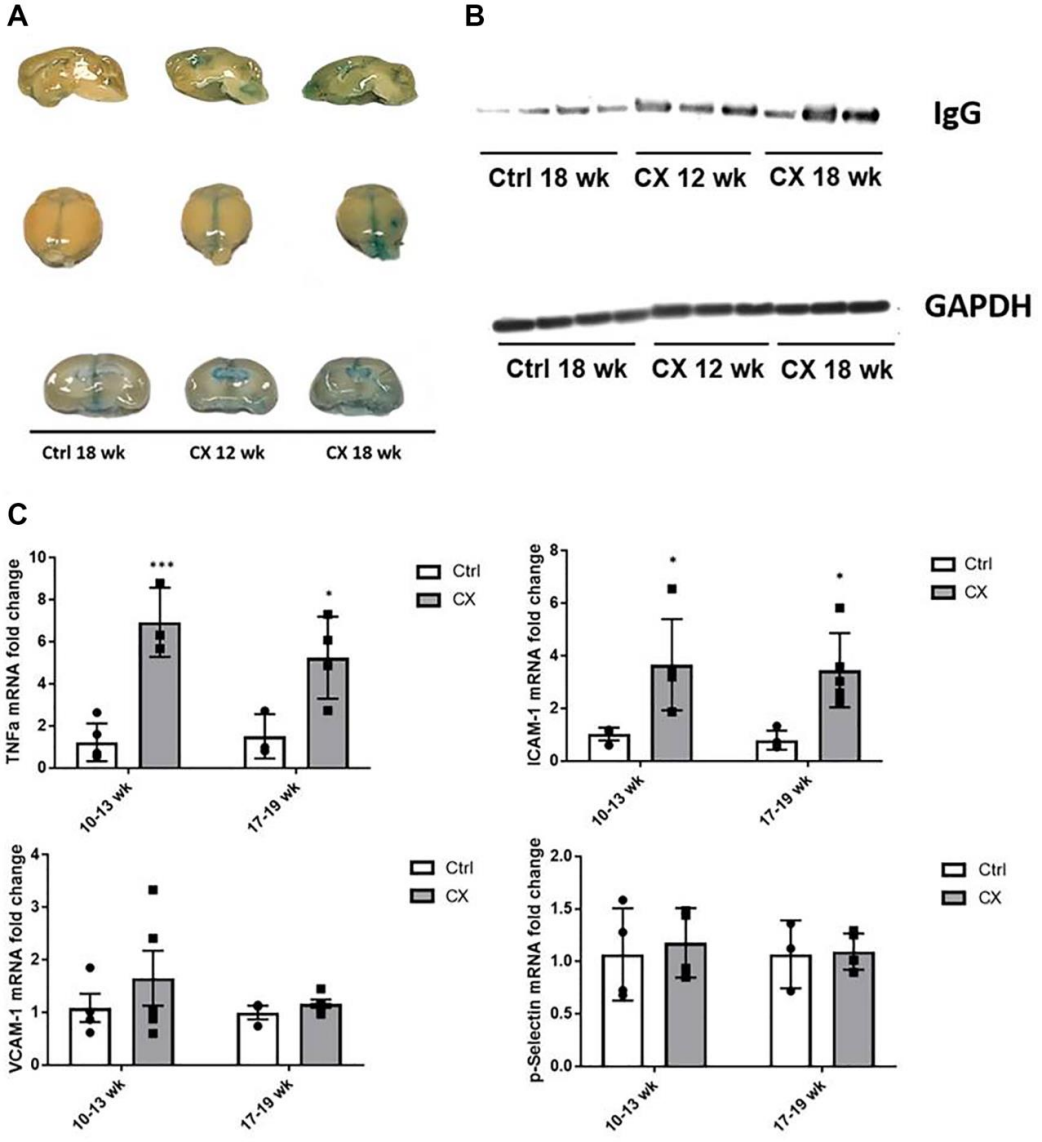
**CX mice display brain vascular permeability and expression of vascular cell activation genes.** (**A**) Evans blue stain shows higher permeability in CX brains in an age-dependent manner (*n* = 2). (**B**) Western blot of IgG, another marker of permeability of the BBB in mice brain. GAPDH was used as a loading control. (**C**) qRT-PCR of vascular cell activation markers, with ICAM-1 and TNFα in CX mice being significantly higher than control *Csa^−/−^* mice, in both 10–13 and 17–19 week old age groups, though there was no difference between age groups. Data are presented as mean ± SE. Two way Anova followed by Tukey’s post hoc test, *n* ≥ 4. ^*^P < 0.05, ^**^P < 0.01, ^***^P < 0.001 when comparing within the same age group. Abbreviations: Ctrl: control; Wk: weeks.

DNA damage and the NER pathway are also associated with age-related vascular dysfunction [19]. We thus investigated a possible association between the observed central nervous system defects and vascular dysfunction in the CX brain. A feature commonly associated with neurovascular dysfunction is increased permeability of the BBB, which is detrimental to the central nervous system. To assess the integrity of the BBB in CX mice, we injected the mice with Evans Blue dye, an azo dye with a high affinity to serum albumin. Under normal circumstances, albumin cannot cross the BBB due to its high molecular weight. When the BBB is compromised, the albumin-Evans blue complex can enter the brain tissue and stain it. We observed pronounced staining in CX mice brains, increasing in an age-dependent manner (Figure 2A). These observations indicate a neurovascular dysfunction in CX mice brain, which could potentially contribute to its neurodegenerative phenotype. Moreover, we also observed a robust increase in the levels of the IgG protein in CX mice brains, confirming the higher permeability of the BBB compared to the control mouse line (Figure 2B).

Several proteins are commonly associated with vascular activation and inflammation, many of them overlapping. We measured mRNA expression of several of these factors (ICAM-1, TNFα, p-Selectin, VCAM-1) by qRT-PCR, and we found that the levels of ICAM-1 and TNFα gene expression were significantly higher in brains of CX animals (*n* ≥ 4) in both age groups tested (10–13- and 17–19-week-old animals, Figure 2C), indicating neurovascular dysfunction and neuroinflammation in CX mice.

### CX mice do not display cell autonomous vascular dysfunction

**Figure 3:**
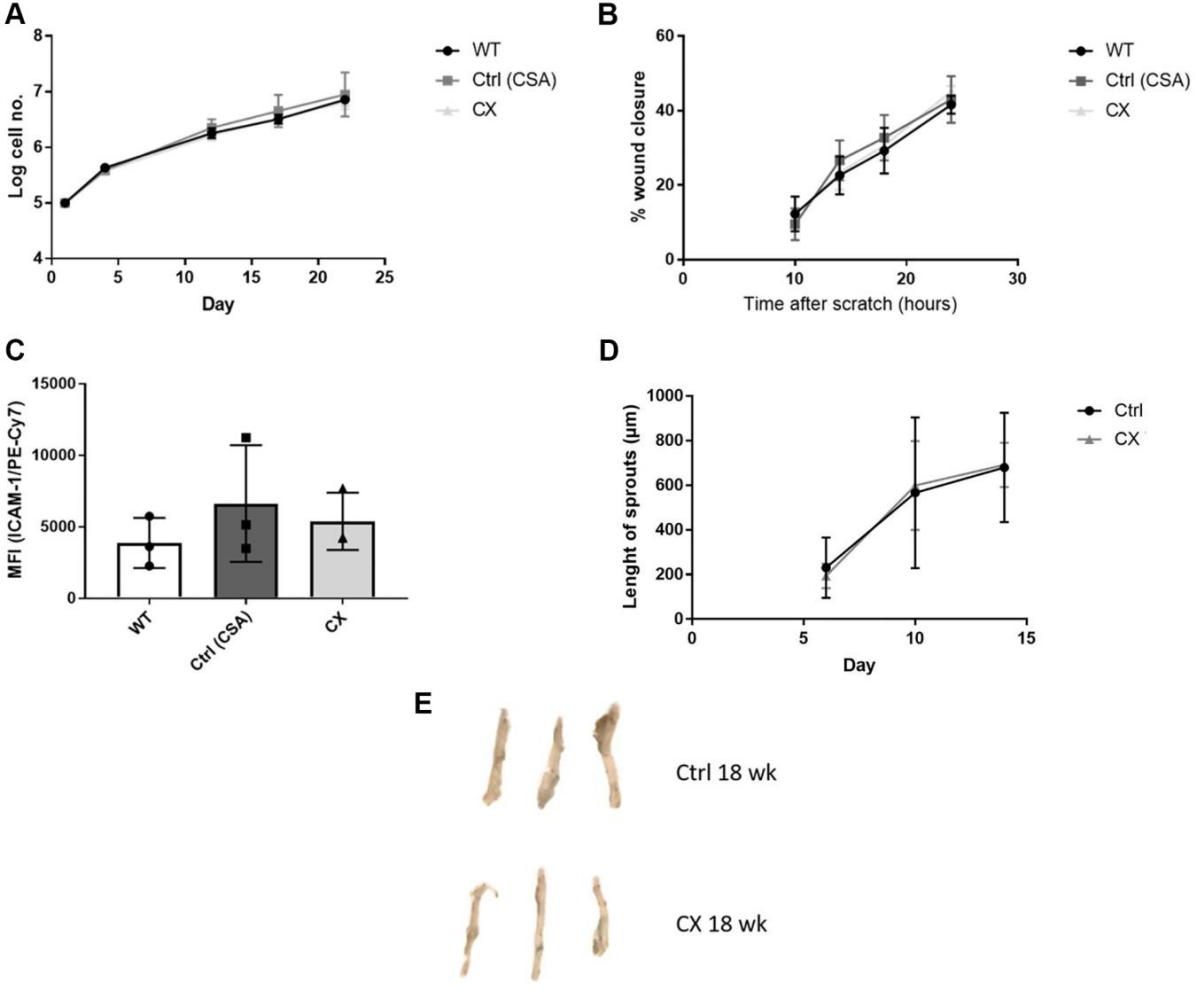
**CX mice do not display cell autonomous vascular dysfunction phenotype regarding endothelial cell proliferation, migration, ICAM-1 activation, angiogenesis nor senescence.** (**A**) Proliferation rates of CX ECs do not differ from neither the CSA KO nor WT ECs. (**B**) Migration capacity, as measured by the wound healing assay. (**C**) ICAM-1 expression in ECs, measured by fluorescence intensity in FACS analysis. One-way Anova followed by Tukey’s post hoc test. (**D**) Angiogenesis capacity, measured using the aortic ring assay in 12 week old animals. Data are presented as mean ± SE. Student’s *t*-test. *p* values of higher than 0.05 were considered nonsignificant. (**E**) Senescence Associated β-galactosidase staining of mice aortas. *n* ≥ 3 for all experiments. Abbreviations: Ctrl: control; WT: wildtype; EC: endothelial cells; Wk: weeks.

To investigate whether EC dysfunction plays a role in the CS phenotype, we generated primary ECs lines from WT, CSA KO, and CX mice to study cell proliferation, migration capacity, and EC activation in a cell autonomous manner. We also performed the aortic ring assay, an *ex vivo* assay to measure angiogenesis, and investigated EC senescence by measuring Senescence-Associated-βeta-galactosidase (SA-β-gal) staining of mouse aorta. We did not detect any differences between any of the genotypes regarding proliferation (Figure 3A), migration (Figure 3B), or ICAM-1 activation (Figure 3C) in isolated ECs (*n* = 3), which indicate that, in contrast to other similar progeroid models, CX mice do not display *in vitro* EC dysfunction. Furthermore, we also found no decrease in angiogenesis capacity (Figure 3D) or premature senescence (Figure 3E, Supplementary Figure 4) of CX mice aortas (*n* = 3).

### Senescence and pro-inflammatory markers in CX mice brains

**Figure 4:**
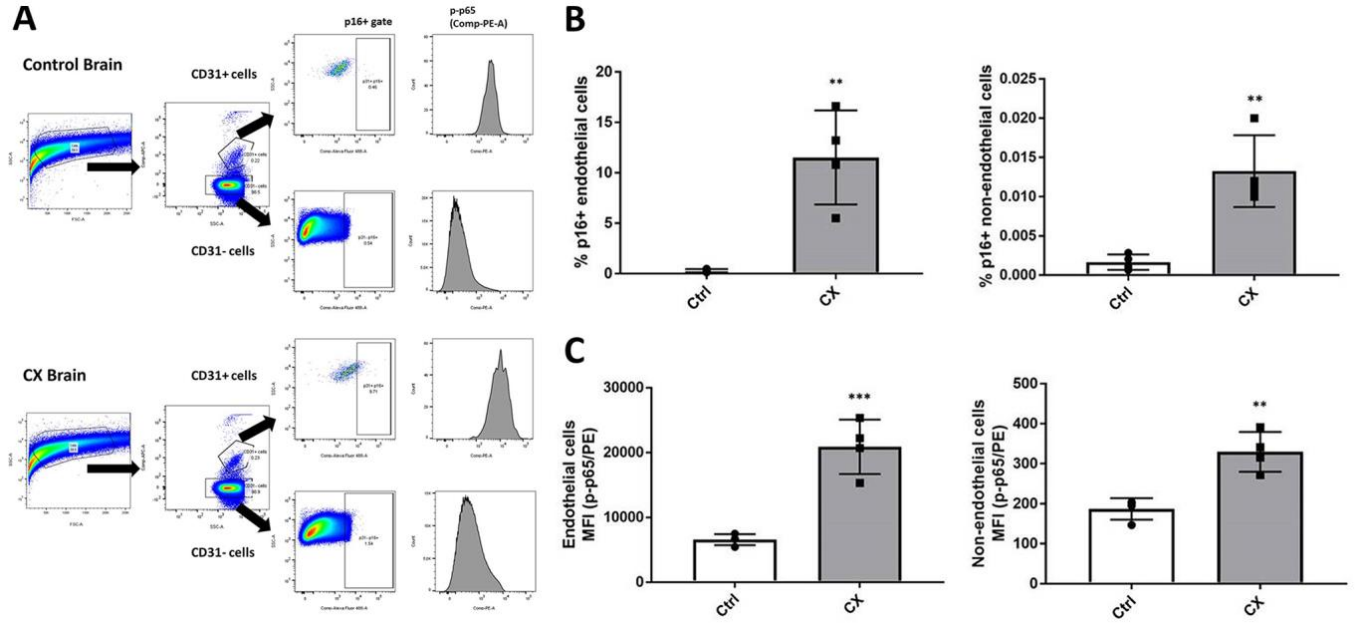
**CX mice brain display p16^Ink4a^ and p-p65 upregulation in endothelial and non-endothelial cells.** (**A**) Flow cytometry gating strategy for the detection of senescent and pro-inflammatory endothelial and non-endothelial cells. Whole brain cells were stained for EC marker CD31, senescence marker p16^Ink4a^, and active NF-κB (phosphorylated p65). Brain cells were first identified using a forward scatter (FSC) and side scatter (SSC) gate. CD31 positive and negative cells were then identified by their APC fluorescence levels. p16+ senescent cells were then identified using an SSC and Alexa Fluor 488 gate. Phosphorylated-p65 expression in CD31+ and CD31- cells was measured by analyzing PE median fluorescence intensity. (**B**) FACS analysis of endothelial and non-endothelial p16^Ink4a^ positive cells in CX mice brains. (**C**) Phosphorylated p65 FACS analysis of endothelial and non-endothelial cells in CX mice brains, *n* = 4. Data are presented as mean ± SE. Student’s *t*-test. ^*^P < 0.05, ^**^P < 0.01. Abbreviation: Ctrl: control.

Although we did not find any evidence of cell autonomous EC dysfunction or senescence, we also investigated this phenotype in the brain of CX mice, as it is one of the most notably affected organs of CS patients. Senescence is a process heavily influenced by the extracellular milieu, often occurring in a tissue-specific manner. Senescent cells also contribute to inflammation by developing a pro-inflammatory senescence-associated secretory phenotype. To investigate whether brain ECs have these phenotypes, we used an *ex vivo* strategy, in which we processed the brain into a single cell suspension by physical and chemical dissociation, then immunostained ECs using anti-CD31, (an EC marker), p16^Ink4a^ (a senescence marker), and p-p65 (a marker of active/pro-inflammatory NF-κB). Samples were then analyzed using flow cytometry through the gating strategy outlined in Figure 4A. We found a significant upregulation of p16^Ink4a^ and p-p65 in both CD31 positive cells (Figure 4B) and CD31 negative cells (Figure 4C) of 18-week-old (*n* = 4) CX mice brain. These results indicate an early senescence phenotype in CX brain ECs *in vivo* which is not seen in cell autonomous conditions, as well as neuroinflammation in the brain of these mice. In contrast to other similar NER-deficient models [30], CX serum did not show a significant increase of pro-inflammatory factors TNFα or IL-6 (Supplementary Figure 5), suggesting that the observed brain inflammation is likely a tissue specific phenotype.

### Glial cell activation in CX mice brain

**Figure 5:**
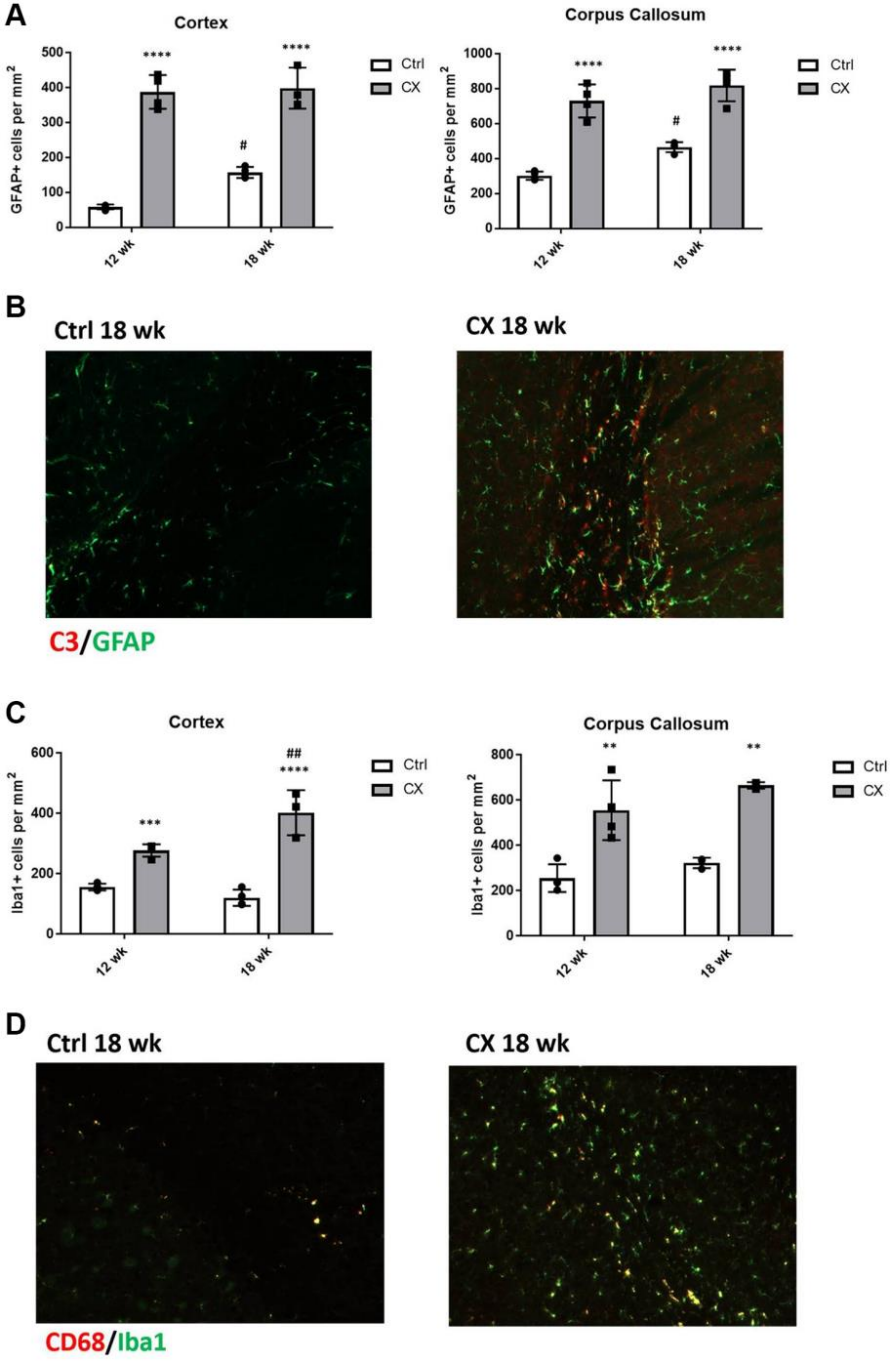
**CX mice display astrocyte and microglia activation in the brain.** (**A**) Reactive astrocytes number increase in CX brain cortex and corpus callosum, as measured by GFAP staining. (**B**) C3, a marker of active astrocytes co-stains with GFAP in CX mice. (**C**) Microglia number in CX brain cortex and corpus callosum, shown by Iba1 positive cells. (**D**) CD68, a marker or active microglia co-stains with Iba1 in CX mice brain, *n* = 4 for all experiments. Data are presented as mean ± SE. Two way Anova followed by Tukey’s post hoc test, *n* = 4. *P* values of less than or equal to 0.05, 0.01, 0.001 and 0.0001 are indicated by asterisks (^*^) when comparing within the same age group and by the pound sign (^#^) when comparing between age groups. Abbreviations: Ctrl: control; Wk: weeks.

We next investigated whether the two glial cell types involved in neuroinflammation, astrocytes, and microglia, displayed abnormal activation status and increased numbers in CX mice compared to the control mice (*n* = 4). We observed a marked increase in the number of GFAP^+^ activated astrocytes (Figure 5A) and C3 staining (Figure 5B) in CX mice, which was maintained with age. We also found that the number of microglia in CX brain is increased in an age-dependent manner (Figure 5C), and that these cells are in a more active state when compared to control mice, as observed by Iba1 and CD68 staining (Figure 5D).

### Impairment of neovascularization does not increase neuroinflammation markers in adult CX mice

**Figure 6:**
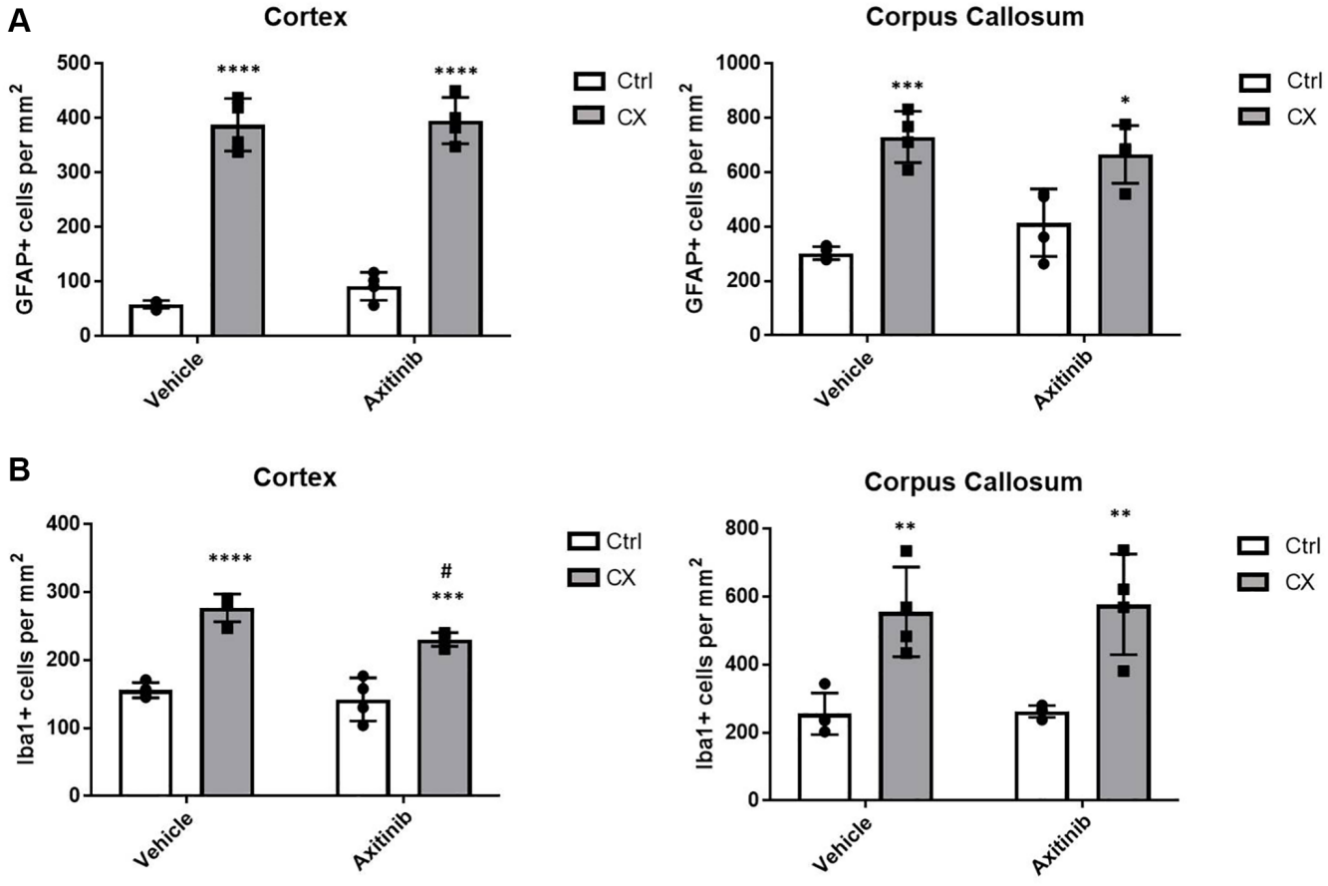
**Impaired angiogenesis does not increase astro or microgliosis in adult CX mice.** (**A**) Axitinib treatment does not alter astrogliosis in adult CX brain cortex nor corpus callosum, as measured by GFAP staining. (**B**) Axitinib treatment decreases microgliosis in the cortex, but not the corpus callosum of CX mice. Data are presented as mean ± SE. Two way Anova followed by Tukey’s post hoc test, *n* = 4. *P* values of less than or equal to 0.05, 0.01, 0.001 and 0.0001 are indicated by asterisks (^*^) when comparing within the same treatment group and by the pound sign (^#^) when comparing between treatment groups.

To test whether angiogenesis impairment, a marker of vascular dysfunction, would increase brain inflammation in these animals, we used older, post- developmental stage animals (9 weeks old), and treated them for 3 weeks with axitinib, an angiogenesis suppressor that inhibits VEGFR2. Surprisingly, we found no increase in the astro- or microgliosis markers in axitinib treated animals (Figure 6, *n* = 4), suggesting that reduced angiogenesis is not a primary source of the neuroinflammation observed in the CX animals. Instead, we found a slight decrease of microgliosis in CX brain cortex, possibly due to inhibition of downstream VEGFR2 pro-inflammatory signaling pathways, such as p38 MAPK or ERK [31].

Curiously, axitinib treatment caused premature death in young (4–6-week-old) CX mice (Supplementary Figure 6), implying that CX mice require VEGFR2 signaling during postnatal development. As NER-deficient mouse pups present reduced levels of several growth factors [13, 32], the pharmacological inhibition of VEGFR2 may impair the survival of developing CX mice.

## DISCUSSION

Cockayne syndrome is a multifactorial disease that affects multiple organ systems, with neurodevelopmental and neurodegenerative defects among the most prominent phenotypes [33]. Although mutations in DNA repair-related genes cause CS, it is still unknown what role DNA damage plays in the etiology of the progeroid phenotype presented by CS patients [6]. The proteins encoded by CS-related genes have several functions besides transcription coupled repair, such as chromatin remodeling, ribosomal biogenesis, and gene regulation [34, 35]. In this work, we used a double knockout *Csa*^−*/*−^*/Xpa*^−*/*−^ genetic mouse model that mimics various progeroid CS phenotypes [16], including those affecting the central and peripheral nervous system.

Reports using other similar NER-deficient mouse models have suggested that vascular dysfunction plays a role in the reduced lifespan and other progeroid features of these models. Indeed, Ercc1^Δ/−^ mice display an age-dependent endothelial dysfunction, such as increased blood pressure [36] and early *in vivo* senescence of thoracic aorta, albeit with no changes in angiogenesis potential from aortic explants. [19]. Ercc1^Δ/−^ ECs also presented early proliferative senescence *in vitro* [19]. Endothelial-specific gene deletion of *Ercc1* also severely limits mouse lifespan and causes progressive vascular dysfunction, such as altered end organ perfusion [37]. We thus sought to investigate whether the CX model also displayed endothelial dysfunction, as well as its possible role in the observed neurodegenerative phenotype.

However, EC lines obtained from CX mice did not present any *in vitro* differences regarding cell proliferation, migration, or ICAM-1 activation compared to control ECs. In addition, CX aorta did not display early senescence nor decreased angiogenesis potential *ex vivo*, indicating that ECs do not play a role in a cell autonomous manner regarding the CX phenotype. Previous findings have demonstrated that CX mice present a higher production of hydrogen sulfide (H_2_S) both *in vivo* and in cultured ECs, as an adaptive response to DNA damage [38]. As H_2_S is crucial for age-related vascular health [39], this increase in H_2_S might explain the lack of cell autonomous EC dysfunction in CX mice. Furthermore, we found no increase of pro-inflammatory factors TNFα and IL-6 in the serum of CX mice, which indicates that this model does not exhibit systemic inflammation nor EC activation. Nevertheless, local factors such as the presence of senescent perivascular fat cells or other pro-inflammatory cells residing in a close proximity to the ECs could still generate a local vascular dysfunction phenotype [40].

Despite having no cell autonomous EC defects, we found higher brain vascular permeability in CX mice, which indicates BBB dysfunction in this progeroid model. CX brain also show an increase in the expression of ICAM-1, TNFα, and active NF-κB, markers of vascular activation and inflammation [41], alongside an increase in p16^Ink4a^ positive cells, a marker of senescence [42]. Taken together these results suggest that CX mice have a brain EC dysfunction. We hypothesize that pro-inflammatory factors, as indicated by the higher levels of TNFα and active NF-κB, influence the brain microenvironment in a neuroinflammatory, deleterious manner, and thus could be involved in the brain vascular dysfunction also observed in other similar neuropathologic models [43, 44].

Furthermore, we found an increase in reactive pro-inflammatory astrocytes and microglia, a phenotype also found in other progeroid models, in CS patient cerebella, and during pathologic and normal ageing [15, 35, 45]. The observed astro- and microgliosis is presumed to be independent of the neurovascular dysfunction phenotype, as neovascularization impairment did not augment this phenotype. The relationship between astrocytes, microglia, neuroinflammation, and neurodegeneration is a very complex one, as these multifunctional, versatile glial cell types are involved in a variety of processes, such as energy metabolism, debris clearance, synaptic maintenance, cell signaling, and regulation of anti and pro inflammatory stimuli [46]. Therefore, it is important not to overstate the effects of an increase in the number of reactive glia, as there are numerous functions that these cells could be carrying out. For instance, since astrocytes are the main cells that provide energy to brain cells, especially neurons [47], the observed astrogliosis in CX mice could also be associated with the necessity of these cells to meet the energy requirements of the other brain cells, as CX mice have a higher fatty acid oxidation rate and generally more active metabolism [28].

Nevertheless, considering the active pro-inflammatory characteristics of glial cells, these phenotypes are correlated to numerous neurodegenerative diseases. Astrocytes and microglia with this activation state can induce neuronal and oligodendrocyte cell death and synaptic and cell signaling dysfunction [23, 26]. The results show that CX mice have a more pronounced gliosis, and also that these glial cells are in an active state, as observed by the expression of C3 in astrocytes and CD68 in microglia, markers of a toxic activation state found in other neurodegenerative models [26, 48]. Moreover, other models associate pro-inflammatory stimuli by glial cells with higher BBB permeability and dysfunction [49, 50]. We thus hypothesize that CX astrocytes and microglia play causal roles in the neuroinflammation, neurodegeneration, and brain vascular dysfunction observed in the central nervous system of these animals.

As both Csa and Xpa proteins are necessary for TC-NER, it stands to reason that the overt neurodegenerative phenotype observed in the CX model compared to single knockout *Csa*^−*/*−^ or *Xpa*^−*/*−^ mice is due to a deficiency in other, non-overlapping functions performed by these proteins, in addition to the lack of TC-NER. Interestingly, both CSA and XPA proteins act in other DNA damage responses [51–54], and PARP-1, a NAD^+^-dependent Poly(ADP-ribose) polymerase involved in multiple DNA repair pathways [28, 55], was found to be upregulated in CX nervous tissue. It is therefore possible that increased accumulation of DNA damage is the underlying cause of the more overt phenotype observed in the CX model, a notion previously proposed to explain similar double knockout NER-deficient mouse models, namely the *Csb^m/m^/Xpa*^−*/*−^ [15, 17, 56] and *Csb^m/m^/Xpc*^−*/*−^ models [56, 57].

Furthermore, CX brain tissue presented reduced NAD^+^ levels, possibly as a result of overt PARP-1 activation, a feature also found in other DNA repair deficient models [58, 59]. NAD^+^ signaling is integral to mitochondrial homeostasis, involved in mitophagy through the NAD^+^-SIRT1-PGC1α axis [27]. Impairment of this axis has been found in cells derived from XP-A patients [60], as well as in several CS models [61], including CX mice [60]. Defective mitophagy is emerging as a major factor in neuroinflammatory diseases [62], as accumulation of damaged mitochondria causes the release of damage-associated molecular patterns, which in turn can activate pro-inflammatory pathways such as (cGAS)-STING [63] and the NLRP3 inflammasome [59]. Interestingly, these pathways are also associated with the upregulated neuroinflammatory markers observed in CX brain [4, 63–65].

However, while CX mice exhibited an increase in brain mRNA expression of ICAM-1 and TNFα, these transcripts can also be regulated at translational and post-translational levels [66, 67], and may be acting in a specific brain region [68, 69]. Moreover, although CX brain cells displayed increased active NF-kB, processes other than defective mitophagy might be the underlying cause of this modulation, as NF-kB may also be activated by numerous other stimuli, including as a response to DNA damage [65, 70]. Therefore, elucidating the molecular mechanisms behind this CS-related glial cell activation and neuroinflammation could enable further insight into this multifactorial disease's etiology, thus providing novel therapeutic targets for the treatment of this syndrome.

## MATERIALS AND METHODS

### Mice lines

Knockout (KO) mice of the *Csa* and *Xpa* genes and double KO (CX) strains have been described previously, with genotyping, care and housing of the mice strains performed as described [16]. Briefly, to perform genotyping of the CX colony, DNA was extracted by boiling mice ear punch tissue at 100°C with 50 mM NaOH, followed by neutralization with 1 M Tris-HCl. PCR was performed as per Supplementary Tables 1–3. Expected band sizes for wild type and knockout alleles for *Xpa* genotyping are 300 bp and 200 bp, while for *Csa* they are 230 bp and 140 bp, respectively. All strains used in this study (WT, CSA KO, XPA KO and CX) had a C57BL6/J background. Animals were maintained by crossing *Csa*^−/−^/*Xpa*^+/−^ with *Csa*^−/−^/*Xpa*^+/−^ to obtain *Csa*^−/−^/*Xpa*^−/−^ CX animals. *Csa*^-/-^*/Xpa*^+/+^ mice were regarded as controls (Ctrl) for all experiments in this study. Housing, breeding, and experimentation were performed in accordance with the regulations established by the Harvard Medical Area Institutional Animal Care and Use Committee (IACUC). Animals were fed using D12450B (Research Diets, New Brunswick, NJ) as previously described [16].

### Pharmacological interventions

Axitinib (VEGFR2 specific inhibitor) was obtained from Selleckchem (#S1005) and supplemented at a daily dose of 30 mg/kg/d in food (Research Diets D12450Bpx), a dose previously described as having anti-angiogenic effects in mice [71].

### Brain vascular permeability

To assess the permeability of the BBB, mice were injected intravenously with 200 μl of PBS with 2% Evans blue (Sigma-Aldrich, St. Louis, MI, USA), sacrificed after 1 h, and perfused intracardially with PBS. Brains were then harvested and sectioned to investigate the inclusion of Evans blue into the brain tissue.

### IgG western blot

IgG infiltration, another marker of BBB permeability [72], was measured via western blot in order to assess the presence of this protein in mice brains. Brain protein was isolated by grinding tissue in NP-40 buffer containing protease inhibitors and dithiothreitol (DTT). After isolation, protein concentration was quantified and normalized using Pierce BCA Protein Assay Kit (Thermo-Fisher, Waltham, MS, USA), boiled in sodium dodecyl sulfate (SDS) buffer and separated through polyacrylamide gel electrophoresis. Proteins were transferred to polyvinylidene difluoride membranes and blotted for mouse IgG (P 0447, Dako, Santa Clara, CA, USA) and GAPDH (sc-137179, Santa Cruz Biotechnology, Dallas, TX, USA).

### Gene expression analysis by qPCR

RNA was isolated from brain tissue with RNA bee (Invitrogen, Life Technologies, Carlsbad, CA, USA) according to the manufacturer’s protocol, with tissue being grinded in RNA bee on ice, followed by ethanol washes. RNA was precipitated with isopropanol, and quantified with a Nanodrop 2000 spectrophotometer (Thermo Fisher). cDNA was synthesized using 1 μg of total RNA using the Verso cDNA kit according to manufacturer instructions (Thermo Fisher). qRT-PCR was performed with SYBR green master mix (BIORAD, Hercules, CA, USA). Fold changes were calculated by the ΔΔCt method using β-actin or GAPDH as standard, and normalized to the experimental control. Primer sequences are available in Supplementary Materials.

### Primary endothelial cell culture

Endothelial cell lines were obtained from lungs of WT, CSA KO and CX mice strains to assess EC proliferation capacity, migration and ICAM1 activation *in vitro*. For the establishment of the cell lines, animals were anesthetized with isoflurane and euthanized by cervical dislocation, followed by the removal of the lungs. The lungs were mechanically dissociated, followed by chemical digestion (200 U/ml Collagenase type II, 200 U/mL Collagenase type IV, 1U/mL Dispase in DMEM) to create a single cell suspension. ECs were purified using the EasySep Mouse APC Positive Selection Kit (Stem Cell Technologies) after incubation with anti-CD31-APC antibody, as per manufacturer instructions. Cells were seeded at a density of 1 × 10^5^ cells/mL in Vasculife complete medium (Lifeline Cell Technology, Frederick, MD, USA). Cultures were incubated in humidified atmosphere with 5% CO_2_ and 5% O_2_. Cells were lifted after reaching approximately 80% confluency by incubation with 0.25% trypsin and counted using a hemocytometer. At least 3 distinct cell lines were used per group per experiment.

### Wound healing assay

To measure migration capacity of ECs, a single scratch wound was created using a sterile p200 pipette tip on a confluent field of cells, 24 h after seeding (100,000 cells per well in 24-well plate) in a serum-free condition. Repopulation across the scratch wound was recorded by a phase-contrast microscopy for up to 48 h using a digital camera. Wound closure was determined at each time point from digital images using ImageJ software.

### Aortic ring assay

To assess the angiogenic capacity of CX mice, we performed the aortic ring assay according to a published protocol [73]. Briefly, we obtained ∼0.5 mm wide rings from mice thoracic aortas, which were then embedded in 50 μL of growth factor reduced Matrigel (Corning) in a 96-well plate. Vessel sprouting was stimulated by complete Vasculife media. The media was replaced every two days, and images were taken using a phase-contrast microscope coupled to a digital camera. The length of sprouts originating from aortic rings was quantified by ImageJ software. Aortic rings were collected from 3 mice per group and the assay was performed using 3 technical replicates.

### Tissue senescence staining

Senescence Associated Beta galactosidase (SA-β gal) staining was performed as per manufacturer instructions (Cell Signaling Technologies, Danvers MS, USA, #9860S). Briefly, aortas and perigonadal fat were removed and washed twice with PBS, followed by fixation with fixation solution for 10 min, washed with PBS, and incubated with β-galactosidase staining solution.

### Serum ELISA

Serum was collected and stored in −80°C conditions until the assay was performed. Mouse IL-6 and TNF-alpha Elisa kits (R&D Systems, Minneapolis, MN, USA) were used according to manufacturer instructions. Briefly, each sample was diluted 5 fold and 50 μL of the diluted sample was added to the ELISA microplate along with 50 μL of the ELISA diluent. After a 2 h incubation, each well was washed 5 times, followed by a 2 h incubation with 100 μL of mouse protein conjugate. Wells were then washed again 5 times, followed by 30 min incubation with 100 μL substrate solution, and 100 μL stop solution. Absorbance was measured with a plate reader set to 450 nm and correction to 570 nm.

### Tissue histology

Mice were perfused with Bouin’s fixative and the Harvard Rodent Histopathology Core performed whole body necropsies. Slides of the central and peripheral nervous system were prepared and stained with hematoxylin and eosin, luxol fast blue, and silver stain according to standardized core protocols.

### Immunohistochemistry

Mice brains were harvested and fixed overnight with 4% PFA at 4°C. The tissue was then washed twice with PBS, followed by 30% sucrose in PBS incubation at 4°C until brains sunk. Brains were then embedded in OCT and cut in a cryotome. Mice gastrocnemius muscle was obtained and embedded in OCT, then cut in a cryotome. For immunostaining, 18 μm coronal brain sections and 5 μm muscle sections cut onto Superfrost VWR slides (VWR International). GFAP, Iba1, CD68 and CD31 were immunostained by first fixing the slides with 4% PFA for 10 min, followed by PBS washes. The tissues were then permeabilized with 0.02% Triton-X for 12 min, followed by PBS washes and 1 h blocking by incubating tissues with 1% BSA in PBS with 10% goat serum solution. Slides were then incubated with GFAP, Iba1, CD68 or CD31 primary antibody solution overnight at 4°C. For C3 staining, tissues were fixed with acetone for 10 min, and antigen retrieval was performed using 50% formic acid for 5 min. After incubation with the primary antibody, slides were washed with PBS, followed by 1 h incubation with secondary antibody solution at room temperature. Antibodies can be found in Supplementary Table 4. Images were taken on Axio Observer fluorescence microscope (Carl Zeiss, Oberkochen, Germany), and GFAP or Iba1 positive cells were quantified using ImageJ software.

### Flow cytometry analysis

Flow cytometry experiments were performed on a BD LSRFortessa and analyzed using FlowJo ver.10. For ICAM-1 staining, ECs were detached from plates using Accumax (Innovative Cell Technologies, San Diego, CA, USA) at approximately 70% confluency, followed by wash and 2 h incubation with PE/Cy7 ICAM-1 antibody (Biolegend, San Diego, CA, USA). Cells were then washed and flow cytometry was performed.

For brain tissue flow cytometry, mouse brains were dissociated in RPMI (Mybiosource San Diego, CA, USA) by gentle trituration using 10 mL pipettes, followed by a 30 min incubation in digestion buffer (200 U/mL Collagenase II, 200 U/mL Collagenase IV, 1 U/mL Dispase). This process was performed twice, to create a single cell suspension, which was stained for ECs using APC-CD31 Antibody (Miltenyi Biotec), followed by incubation at 4°C overnight in fixation buffer (eBiosciences). Cells were then washed and incubated in permeabilization buffer (eBiosciences) for 1 h. Brain cells were then stained for p16^Ink4a^ and p-p65 by incubating overnight at 4°C with primary antibody solution, followed by incubation with secondary antibody for 1 h at room temperature. Cells were washed with PBS and incubated with 1% BSA in PBS with 10% goat serum solution for flow cytometry. Antibodies can be found in Supplementary Table 4.

### Transmission electron microscopy

Animals were perfused with fixative (2.5% glutaraldehyde, 1.25% paraformaldehyde, and 0.03% picric acid in 0.1 M sodium cacodylate buffer pH 7.4). Sciatic nerves were isolated and postfixed for 2 h at room temperature in the same fixative, washed in 0.1 M sodium cacodylate buffer and postfixed with 1% osmium tetroxide/1.5% potassium ferrocyanide for 1 h, washed 3× with water and incubated in 1% aqueous uranyl acetate for 1 h followed by 2× wash in water and dehydration in grades of alcohol (10min each; 50%, 70%, 90%, 2 × 10 100%). Samples then put in propyleneoxide for 1 hr and infiltrated overnight in a 1:1 mixture of propyleneoxide and TAAB Epon (Marivac Canada). The following day samples were embedded in TAAB Epon and polymerized at 60°C for 48 hrs. Thick 0.5 μm sections were cut and stained with toluidine blue for assessment and ultrathin sections (60 nm) were cut on a Reichert Ultracut-S microtome, pick up on to copper grids stained with lead citrate and examined in a JEOL 1200EX Transmission electron microscope or a TechnaiG2 and images were recorded with an AMT 2k CCD camera.

### Measurement of NAD^+^ content

Approximately 20 mg size pieces of tissue were prepared and used in the EnzyChrom NAD^+^ Assay Kit from BioAssays (Hayward, CA). Samples were normalized to protein content.

### Fatty acid oxidation capacity

Sciatic nerve and whole brain were removed and split in half for 2× cortex and 2× cerebellum pieces. Tissues were then weighed and placed in KH buffer containing 25 mM NaHCO_3_, 118 mM NaCl, 4.7 mM KCl, 1.2 mM MgSO_4_, 1.2 mM NaH_2_PO_4_, 1.2 mM CaCl_2_ and 2.5 mM glucose. Tissues were kept in buffer on ice until all dissections were completed. Each tissue then transferred to KH buffer plus 2% Fatty acid free BSA and 2.5 mM glucose, with 2 μCi 3H palmitic acid (Perkin Elmer, Waltham, MA, USA) and incubated at 37°C for 1 hr. The buffer was collected and hydrolyzed 3H palmitic acid (as 3H water) was extracted. 100 μL of buffer was added to 100 μL of 10% trichloroacetic acid (TCA), vortexed, incubated at RT for 15 min, spun at 16,000 rpm for 10 min, and the supernatant collected into a new tube. TCA (5%; 100 μL) and 40 μL BSA (10%) was added to the supernatant, vortexed, incubated at RT for 15 min, spun at 16,000 rpm for 10 min, and the supernatant transferred to a new tube. Chloroform:methanol (2:1, 750 μL) was added to the supernatant, along with KCl: HCl (2 M each, 300 μL), vortexed and spun at 16,000 rpm for 10min. The upper layer (~600 μL) was collected into 5 ml EcoLume, mixed, and counted in a liquid scintillation counter. After subtracting background cpm, the sample cpm was divided by the tissue weight to determine fatty acid oxidation capacity.

### Ketone bodies content assay

β-hydroxybutyrate levels were measured in homogenized tissue using β-Hydroxybutyrate reagent set as per manufacturer instructions (Pointe Scientific, Canton, MI, USA). Tissue was normalized to protein content by BCA.

## Supplementary Materials

Supplementary Materials

Supplementary Figures

Supplementary Tables
