# Supplementary material for: Neurovascular dysfunction and neuroinflammation in a Cockayne syndrome mouse model
Source: Aging (Albany NY). 2021 Oct 10;13(19):22710–31. doi: 10.18632/aging.203617 (PMC8544306; doi:10.18632/aging.203617)
Supplement: Supplementary Materials [file aging-13-203617-s001.pdf]

SUPPLEMENTARY MATERIALS

Primer sequences

Atp5b F:5′-GGGTCAGTCAGGTCATCAGC-3′; R: 5′-CACAATGCAGGAAAGGATCA-3′;
Sca10 F: 5′-CATCTCATCCGTCTGATTGG-3′; R: 5′-GCTGTCCAGGATCAGAGGAA-3′;
Sosc2 F: 5′-CGCGAGCTCAGTCAAACAG-3′; R:5′-AAGAAAGTTCCTTCTGGAGCC-3′;
Nell2 F: 5′-GATGGCTGTAGAAACGGAGG-3′; R: 5′-CCCTCAGAGCACTCGTCAAT-3′;
Peg10 F: 5′-CAGAACGAATAAGGTCCCCA-3′; R: 5′-CATAGCTCGGACAAACAGGG-3′;
β-actin F:5′-AGCTTCTTTGCAGCTCCTTCGTTG-3′; R: 5′-TTCTGACCCATTCCCACCATCACA-3′;
GAPDH F: 5′-AACTTTGGCATTGTGGAAGG-3′; R: 5′-ACACATTGGGGGTAGGAACA-3′;
ICAM-1 F:5′-GCCTCCGGACTTTCGATCTT-3′; R: 5′-GTCAGGGGTGTCGAGCTTTG-3′;
P-Selectin F: 5′-CCCTGGCAACAGCCTTCAG-3′; R: 5′-GGGTCCTCAAAATCGTCATCC-3′;
Tnfα F: 5′-AGGGTCTGGGCCATAGAACT-3′; R: 5′-CCACCACGCTCTTCTGTCTAC-3′;
VCAM F: 5′-AGTTGGGGATTCGGTTGTTCT-3′; R: 5′-CCCCTCATTCCTTACCACCC-3′;
Acox1 F: 5′-CCTGATTCAGCAAGGTAGGG-3′; R: 5′-TCGCAGACCCTGAAGAAATC-3′;
Cd36 F: 5′-GAGCAACTGGTGGATGGTTT-3′; R: 5′-GCAGAATCAAGGGAGAGCAC-3′;
Cpt1c F:5′-CAAACTCTTCCCACCAGTCG-3′; R: 5′-GCAAATGACTTCCTGAGGTTG-3′;
Lcad F:5′-TCTTTTCCTCGGAGCATGACA-3′ R: 5′-GACCTCTCTACTCACTTCTCCAG-3′;
Vlcad F:5′-CTACTGTGCTTCAGGGACACC-3′; R: 5′-CAAAGGACTTCGATTCTGCCC-3′;
Pparα F: 5′-TGTTTGTGGCTGCTATAATTTGC-3′; R: 5′-GCAACTTCTCAATGTAGCCTATGTTT-3′;
Hmgcs1 F:5′-AACTGGTGCAGAAATCTCTAGC-3′; R: 5′-GGTTGAATAGCTCAGAACTAGCC-3′;
Hmgcs2 F:5′-GAAGAGAGCGATGCAGGAAAC-3′; R: 5′-GTCCACATATTGGGCTGGAAA-3′;
