# Supplementary material for: Neurovascular dysfunction and neuroinflammation in a Cockayne syndrome mouse model
Source: Aging (Albany NY). 2021 Oct 10;13(19):22710–31. doi: 10.18632/aging.203617 (PMC8544306; doi:10.18632/aging.203617)
Supplement: Supplementary Figures [file aging-13-203617-s002.pdf]

SUPPLEMENTARY FIGURES

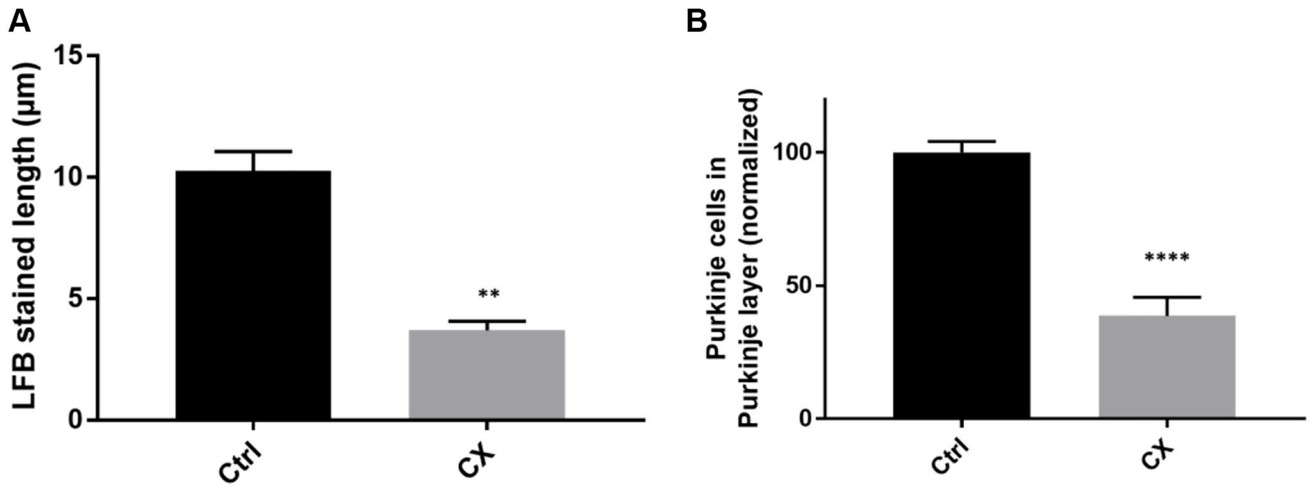

Supplementary Figure 1. Quantification of myelinated areas and Purkinje cell in CX cerebellum. (**A**) Length of LFB stained cerebellum sections of control and CX mice. (**B**) Number of Purkinje cells present in cerebellum Purkinje layer, normalized by control Purkinje cell numbers per layer length.

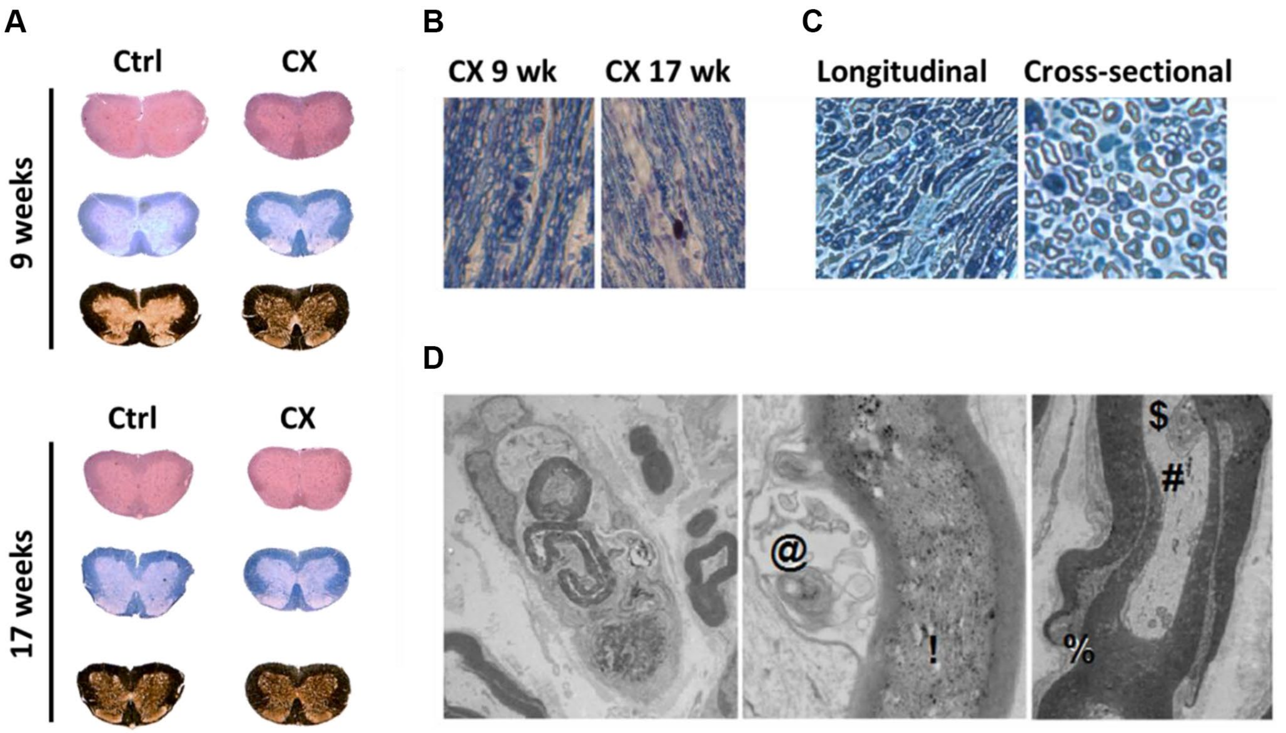

Supplementary Figure 2. Spinal cord and peripheral nervous system disorders in CX mice. (**A**) Hematoxylin and Eosin, luxol fast blue, and silver staining of spinal column of control and CX animals at the indicated ages. (**B**) Luxol fast blue staining of CX sciatic nerves at the indicated ages depicting digestion chambers, mag 1500×. (**C**) Longitudinal and cross-sectional sections of CX sciatic nerve cut 0.5 µm thick and stained with toluidine blue and imaged at 60× magnification. (**D**) Ultrathin sections of sciatic nerve cut at 60 nm thickness, middle panel 15000× magnification and left and right panels at 3000× magnification depicting degeneration of the myelin sheath from the inside out surrounding degenerated neutral lipids (!), myelin balloons (@), neurofilament hyperplasia (#), dense cytoplasm of degenerating lipid ($), and splitting of myelin (%).

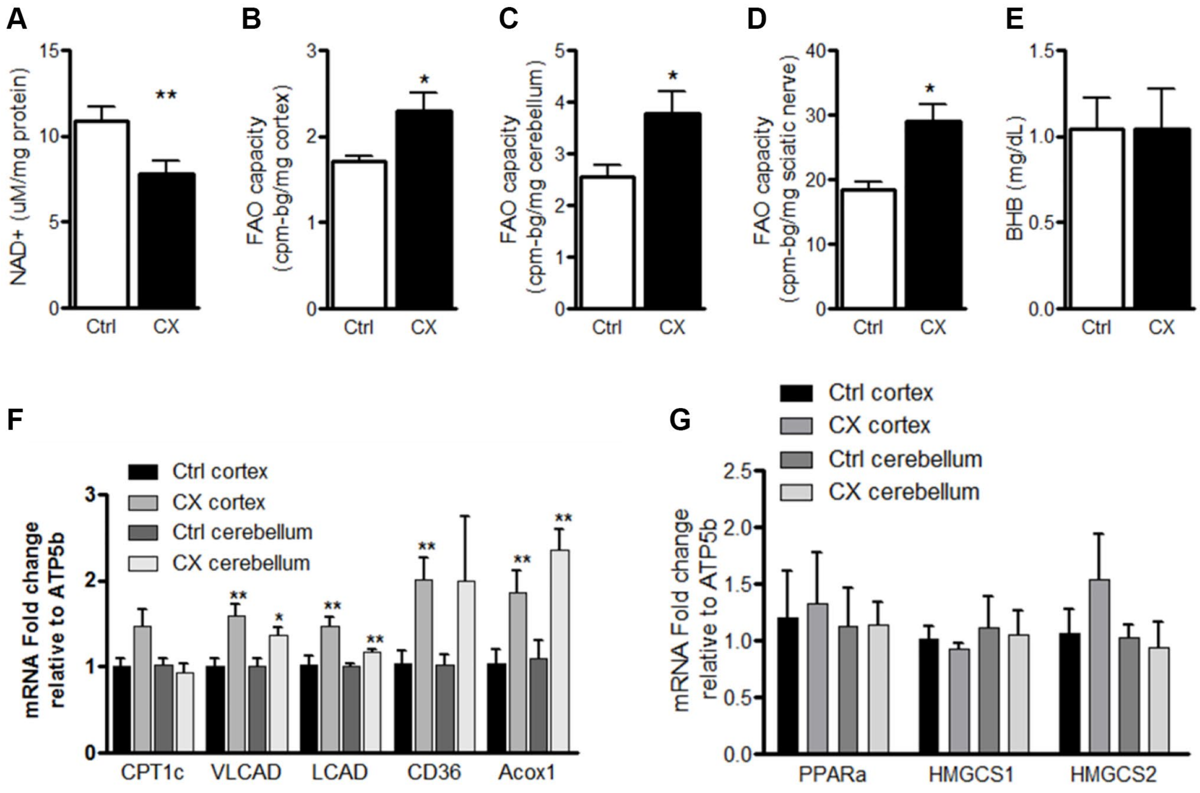

Supplementary Figure 3. Metabolic characteristics of CX central and peripheral nervous system. (**A**) NAD^+ content of whole control and CX brains normalized to protein content, *n* = 9–10 in duplicate per genotype. (**B**–**D**). FAO capacity of control and CX cortex (**B**), cerebellum (**C**), and sciatic nerve (**D**), *n* = 3. (**E**) Beta-hydroxybutyrate levels of control and CX brain lysates, *n* = 3. (**F**) Gene expression of control and CX cortex and cerebellum for FAO related transcripts, *n* = 5. (**G**) mRNA expression of ketogenic genes in control and CX cortex and cerebellum, *n* = 4. Data are presented as mean ± SE. Student's *t*-test. $^{*}P < 0.05$, $^{**}P < 0.01$.

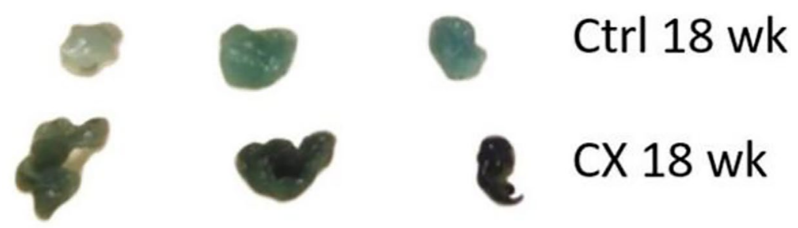

Supplementary Figure 4. Senescence-associated β-galactosidase staining of mice. Perigonadal fat used as a positive control for the SA-βgal assay. Abbreviations: Ctrl: control; wk: week.

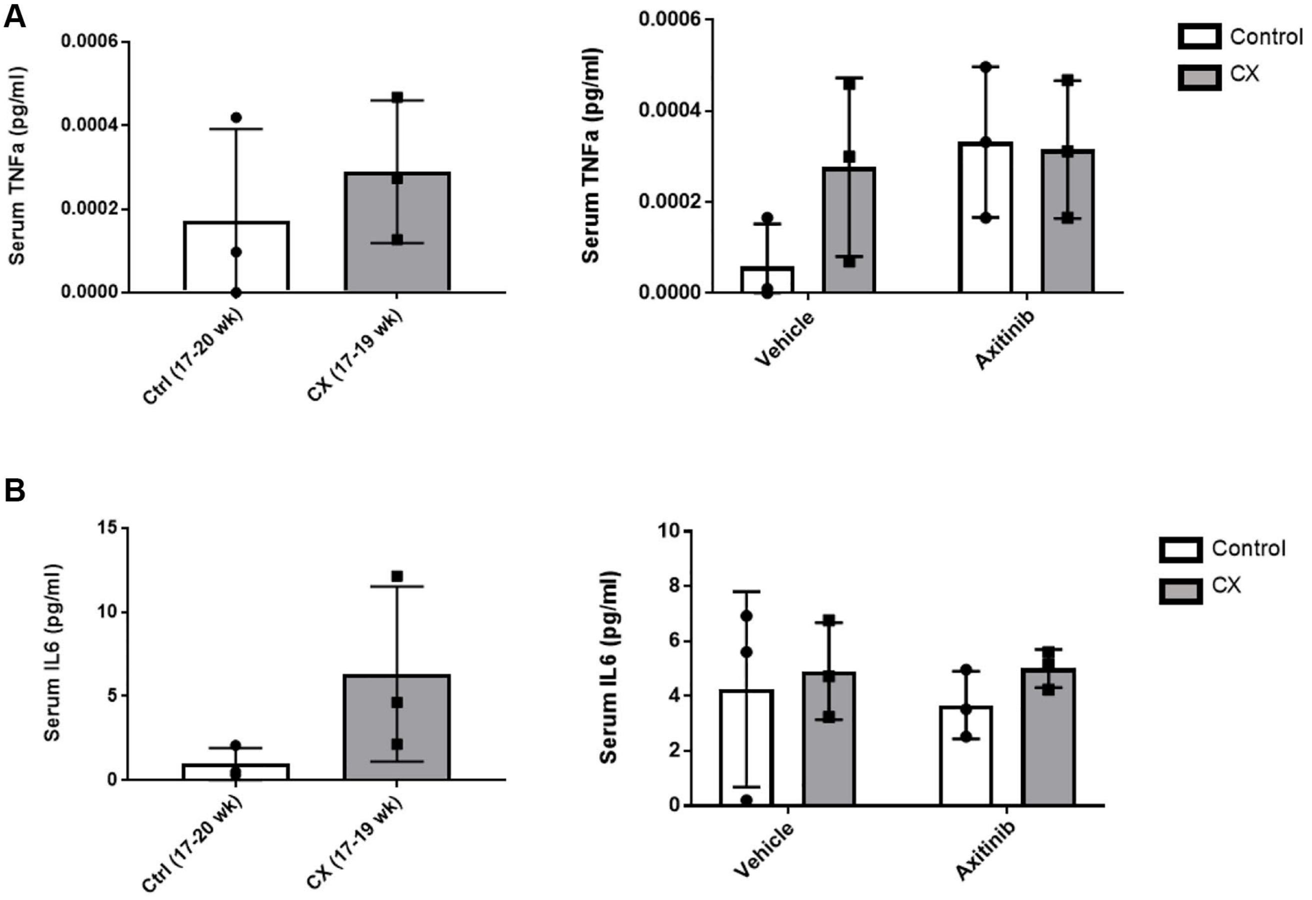

Supplementary Figure 5. CX mice do not have increased serum levels of the pro-inflammatory cytokines TNFα or IL-6. Blood serum of aged CX and control mice, as well as 9–12 week old axitinib treated mice was obtained and used for quantification of TNFα (**A**) or IL-6 (**B**) through ELISA assay. We did not detect any significant levels of either protein in the serum of these mice, indicating that the CX model does not exhibit a general pro-inflammatory phenotype. Data are presented as mean ± SE. *n* = 3. Student's *t*-test.

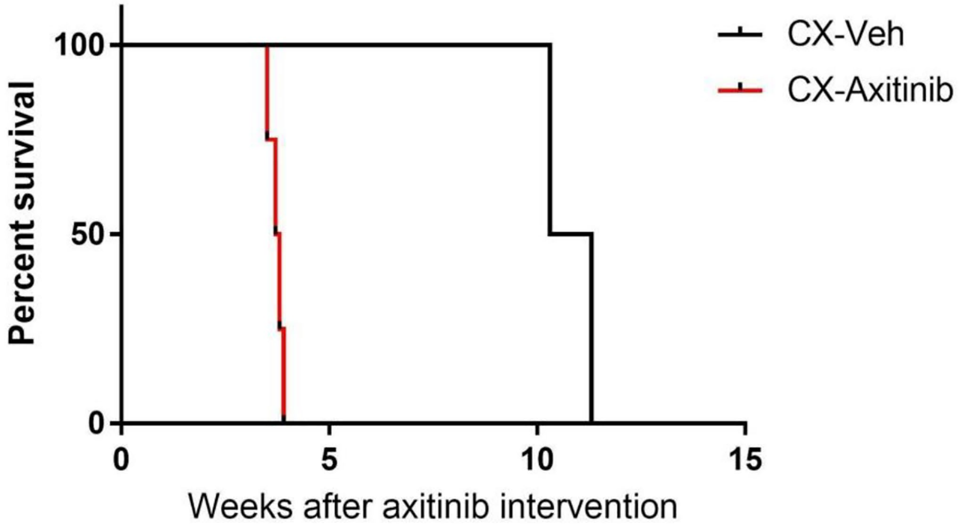

Supplementary Figure 6. Survival of CX mice after axitinib treatment. Kaplan-Meier survival curves of vehicle and axitinib (30 mg/kg/d) treated CX mice, with the treatment starting with ≤ 6 week old animals. *n* = 4.
