# Supplementary material for: Neurovascular dysfunction and neuroinflammation in a Cockayne syndrome mouse model
Source: Aging (Albany NY). 2021 Oct 10;13(19):22710–31. doi: 10.18632/aging.203617 (PMC8544306; doi:10.18632/aging.203617)
Supplement: Supplementary Tables [file aging-13-203617-s003.pdf]

SUPPLEMENTARY TABLES

Supplementary Table 1. Genotyping PCR reagents concentration for *xpa* and *csa* genes.

Target gene	Primers (pM)	DNTPs (pM)	Buffer 10× (μL)	Taq Polymerase (U)	H_2O (uL)
xpa	0.8	0.2	3.75	0.625	q.s. 25
csa	0.66	0.2	3.25	2.5	q.s. 25

Supplementary Table 2. Temperature cycles for genotyping PCRs.

Target gene	Steps												
	1		2		3		4		5	6		7	
	T (°C)	t (s)	T (°C)	t (s)	T (°C)	t (s)	T (°C)	t (s)	Repetitions of steps (2–4)	T (°C)	t (s)	T (°C)	t (s)
xpa	95	120	95	30	62	30	72	60	35	72	300	4	∞
csa	95	300	95	60	62	60	72	60	40	72	600	4	∞

Supplementary Table 3. Primer sequences for genotyping PCRs.

Target gene	Primer	5′->3′ Sequence
	XPA-PGK2 154	GGCCACTTGTGTAGCGCCAA
xpa	XPA26 155	GTGTCAGGCATAAGATCTATGACAA
	XP47 156	AGGCAAGCACCTGCAGCTGT
	CSA6	TCCTGGGGCTGGAGTTAAAC
csa	CSA7	AAAGGCAAGATTTTTCTGCA
	CSA-PGK3	TAGGGGAGGAGTAGAAGGTG

Supplementary Table 4. Antibodies used for immunohistochemistry and flow cytometry analysis.

Antibody	Method	Source	Identifier
Anti-CD68 (FA-11)	Immunohistochemistry	Bio-rad	MCA1957
Anti-Complement C3 (11H9)	Immunohistochemistry	Novus Biologicals	NB200-540
Anti-Iba1	Immunohistochemistry	Novus Biologicals	NB100-1028
Anti-GFAP (GA-5)	Immunohistochemistry	Cell Signaling	3670S
Anti-Goat IgG AlexaFluor 488	Immunohistochemistry	ThermoFisher Scientific	A-11055
Anti-Rat IgG AlexaFluor 568	Immunohistochemistry	Abcam	ab175475
Anti-Mouse IgG AlexaFluor 488	Immunohistochemistry and Flow cytometry	ThermoFisher Scientific	A-21202
Anti-CD31-APC	Flow cytometry	Miltenyi Biotec	130-102-571
Anti-p16INK4a (2D9A12)	Flow cytometry	Abcam	ab54210
Anti-Phospho-NF-κB p65 (Ser536) (93H1) (PE Conjugate)	Flow cytometry	Cell Signaling	5733S
Anti-ICAM-1 (YN1/1.7.4)	Flow cytometry	BioLegend	116122
